# Lewis Acid‐Base Effects on Molecular Structure and Charge Density in Solid Polymer Electrolytes for Solid‐State Batteries

**DOI:** 10.1002/smll.202512202

**Published:** 2026-01-25

**Authors:** Wonmi Lee, Seung‐Min Lee, Min Kyung Kim, Juho Lee, Junhyeok Hwang, Jeongsik Choi, Sungbin Jang, Seok Ju Kang, Sung‐Kyun Jung, Hyun‐Wook Lee, Jinsoo Kim

**Affiliations:** ^1^ Ulsan Advanced Energy Technology R&D Center Korea Institute of Energy Research Ulsan Republic of Korea; ^2^ Department of Chemical Engineering Kongju National University Chungnam Republic of Korea; ^3^ Department of Energy Engineering School of Energy and Chemical Engineering Ulsan National Institute of Science and Technology (UNIST) Ulsan Republic of Korea; ^4^ Department of Nano Fusion Technology Pusan National University Busan Republic of Korea; ^5^ Department of Energy Science and Engineering Daegu Gyeongbuk Institute of Science and Technology (DGIST) Daegu Republic of Korea; ^6^ School of Transdisciplinary Innovations Seoul National University (SNU) Seoul Republic of Korea; ^7^ Research Institute of Advanced Materials (RIAM) Seoul National University (SNU) Seoul Republic of Korea; ^8^ Institute for Rechargeable Battery Innovations Research Seoul National University (SNU) Seoul Republic of Korea

**Keywords:** Lewis acid, Lewis base, charge density, solid polymer electrolyte, solid‐state batteries

## Abstract

Solid polymer electrolytes (SPEs) are critical for advancing the safety and performance of solid‐state batteries (SSBs). However, challenges such as low ionic conductivity, limited mechanical strength, and narrow electrochemical stability windows hinder their widespread adoption. This study investigates the role of lithium salt formulation, specifically lithium bis(fluorosulfonyl)imide (LiFSI), lithium bis(trifluoromethanesulfonyl)imide (LiTFSI), and a binary mixture of the two, on the molecular structure and charge density of poly(vinylidene fluoride‐co‐hexafluoropropylene) (PVDF‐HFP)‐based SPEs. Through comprehensive characterization, we demonstrate that the choice of lithium salt profoundly affects the crystallinity, dehydrofluorination degree, and ionic conductivity of SPEs. Notably, the binary salt mixture provides a balanced improvement in ionic conductivity (4.93 × 10^−4^ S/cm at 20°C) and mechanical strength (127 MPa), alongside a broad electrochemical stability window. These findings suggest that strategic lithium salt formulation can significantly enhance the overall performance of SPEs, paving the way for their effective use in next‐generation SSBs.

## Graphical abstract

Solid polymer electrolytes incorporating LiFSI, LiTFSI, and their binary mixture are investigated to reveal how salt polarity and Lewis basicity regulate dehydrofluorination, crystallinity, and charge density in PVDF‐HFP. The binary‐salt formulation achieves a balanced improvement in ionic conductivity, mechanical robustness, and electrochemical stability, offering a rational strategy to design high‐performance solid polymer electrolytes for solid‐state batteries.

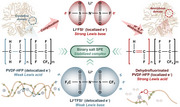

## 1 Introduction

The transition to a more sustainable and electrified future hinges on the development of advanced energy storage technologies. Among these, solid‐state batteries (SSBs) have garnered considerable attention as a promising successor to conventional lithium‐ion batteries (LIBs) [1, 2, 3, 4, 5, 6, 7, 8, 9]. SSBs offer several compelling advantages, including enhanced safety due to the elimination of flammable liquid electrolytes, the potential for higher energy densities through the use of lithium metal anodes, and improved thermal stability. These features position SSBs as a key technology for applications ranging from electric vehicles to grid energy storage, where both performance and safety are paramount.

At the heart of SSB technology lies the solid electrolyte, a critical component that must facilitate the efficient transport of lithium ions while maintaining robust mechanical integrity under operational stresses. SPEs have emerged as strong candidates for this role due to their flexibility, ease of processing, and the ability to form stable interfaces with electrode materials [10, 11, 12, 13]. However, despite their promise, SPEs face several challenges that have slowed their adoption in commercial SSBs. One of the most significant hurdles is the relatively low ionic conductivity of SPEs, especially at ambient temperatures [14]. In traditional liquid electrolytes, the ionic conductivity is typically high due to the mobility of ions in the liquid medium. In contrast, the polymer matrix in SPEs, especially when highly crystalline, can impede the movement of lithium ions, leading to lower conductivity. Enhancing ionic conductivity in SPEs often requires increasing the amorphous content of the polymer, where ion transport is more efficient. However, this approach can negatively impact the mechanical strength of the electrolyte, as amorphous regions are generally less structurally robust than their crystalline counterparts. Balancing ionic conductivity with mechanical strength is further complicated by the need for SPEs to operate within a broad electrochemical stability window [15, 16]. This window defines the voltage range over which the electrolyte remains chemically stable, preventing decomposition that could lead to capacity fade or safety hazards. The challenge, therefore, lies in engineering an SPE that not only facilitates rapid ion transport but also withstands the mechanical and electrochemical demands of a working battery.

Recent research has explored various strategies to address these challenges, including the use of different polymer hosts, the incorporation of plasticizers, and the introduction of various lithium salts [17, 18]. The choice of lithium salt is particularly critical, as it can influence the overall performance through interactions with the polymer matrix. These interactions can affect everything from the degree of polymer crystallinity to the formation of stable complexes that enhance ionic conductivity and mechanical properties. Despite the promising results obtained from different salt formulations, the underlying mechanisms, particularly those related to the polarity and interactions of salts like LiFSI and LiTFSI, are not yet fully understood. These salts have shown potential in various studies, but there remains a need for deeper investigation into how they affect the molecular structure and electrochemical performance of SPEs [19, 20, 21, 22]. The potential differences in their polarity within the polymer matrix, and the implications of these differences on SPE performance, represent a critical area of ongoing research.

In this study, we aim to systematically investigate the effects of these lithium salts, LiFSI, LiTFSI, and their binary mixtures, on the overall performance of PVDF‐HFP‐based SPEs. Rather than focusing solely on their individual properties, we seek to understand how these salts interact with the polymer matrix and each other to influence the key parameters of ionic conductivity, mechanical strength, and electrochemical stability. Through a combination of experimental techniques, including X‐ray diffraction (XRD), Fourier transform infrared spectroscopy (FTIR), and electrochemical impedance spectroscopy (EIS), we will explore how these formulations can be optimized for use in next‐generation solid‐state batteries. As mentioned earlier, several studies have reported that incorporating lithium salts can enhance the properties of solid electrolytes. However, the underlying mechanisms have not been thoroughly discussed. This study introduces the concept of Lewis basicity of lithium salts as a key design parameter for regulating the structure and properties of SPEs. While the concept of Lewis basicity has been applied in the design of liquid electrolytes [23], it has not yet been systematically investigated for SSBs. Our findings will contribute to a deeper understanding of how to design SPEs that meet the stringent requirements of SSBs, providing insights that could accelerate the development of safer, more efficient energy storage systems. This research not only advances the field of SPEs but also provides a foundation for future studies aimed at unraveling the complex relationships between electrolyte composition, molecular structure, and battery performance.

## 2 Results and Discussion

The formulation of SPEs in this study revolves around a carefully selected set of ingredients, each contributing a distinct function to the overall performance of the electrolyte.

**FIGURE 1:**
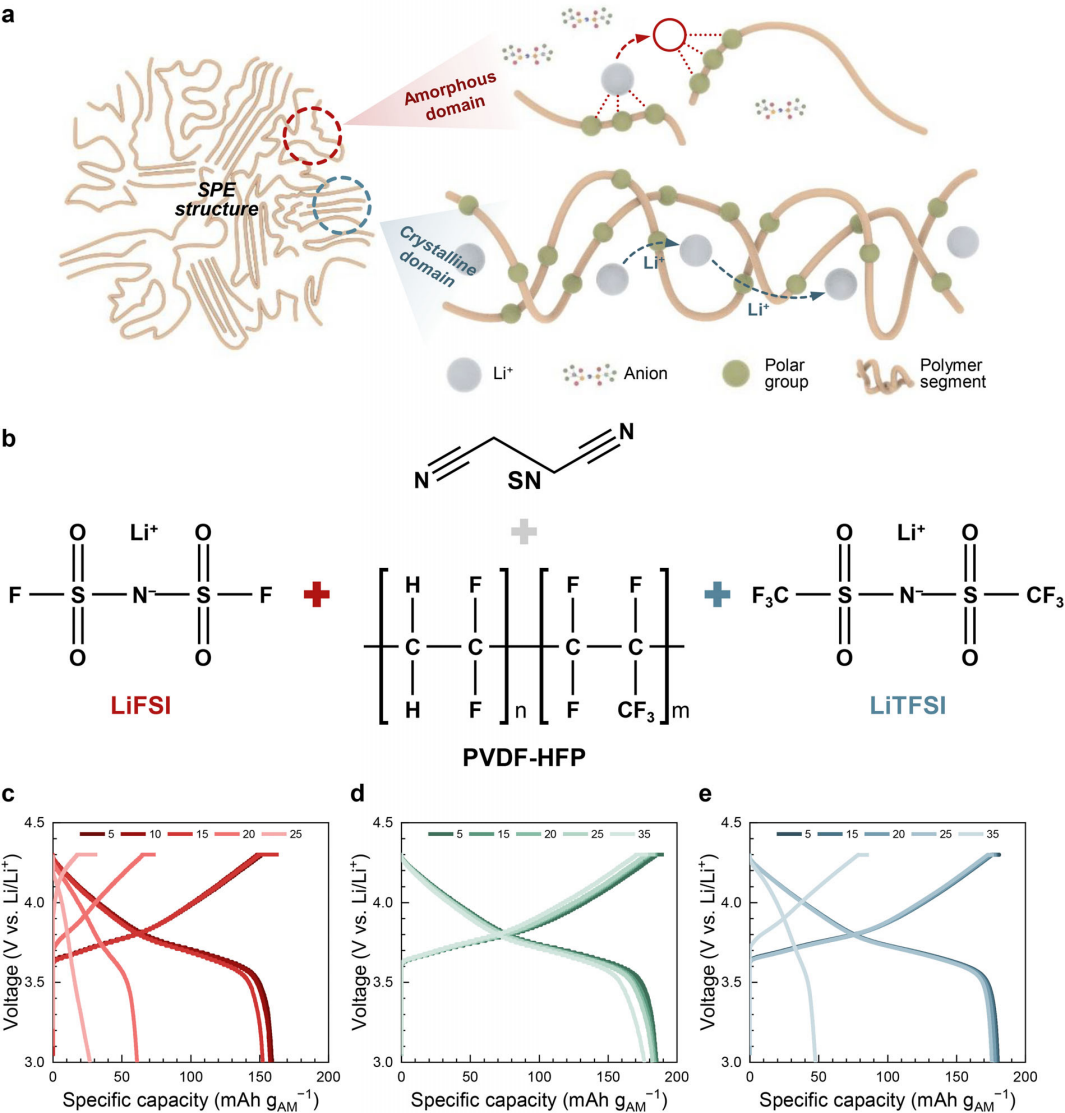
Schematic and cycling performance of SPEs with different lithium salts. (a) Li ion transport mechanism in two distinct domains of SPE. (b) Essential components for SPE formulation: PVDF‐HFP (host polymer), lithium salts (LiFSI, LiTFSI, binary mixture), and SN (plasticizer). Cycling stability of Li/SPE/NCM622 coin cells, comparing SPEs containing (c) LiFSI, (d) their binary mixture, and (d) LiTFSI.

Figure 1a shows the typical lithium ion transporting mechanism of SPEs, which are composed of amorphous and crystalline domains. The amorphous part can easily transport the lithium‐ion by polymer segmental motion aided by its free volume, while the crystalline counterpart is not relatively facile to ion conduction through its functional groups. SPEs with PVDF‐HFP‐based copolymers are regarded as a promising characteristic with their potential functionality and stability [24]. Figure 1b visually summarizes the essential components of those SPEs: PVDF‐HFP serves as the host polymer, providing the structural matrix for the electrolyte [25, 26, 27]. The choice of lithium salt, either LiFSI, LiTFSI, or a binary mixture of the two, is critical in modulating the ionic conductivity and mechanical properties of the SPE [28, 29]. Succinonitrile (SN), included as a plasticizer, enhances the flexibility of the polymer matrix, allowing for better ion mobility while maintaining the mechanical integrity of SPEs [30, 31].

Moving from the formulation to performance, Figure 1c–e, which are also detailed in Figure S1, present the cycling stability of the SPEs when used in Li/SPE/NCM622 (LiNi_0.6_Co_0.2_Mn_0.2_O_2_) coin cells. These figures provide a comprehensive comparison of how the different SPE formulations impact the relative cycling stability and capacity retention over cycles. The cycling performance is not yet satisfactory, indicating that further optimization of the SPE composition and cell configuration is required. However, the primary objective of this study is not to demonstrate optimized electrochemical performance, but rather to elucidate the chemical and structural mechanisms associated with the dual‐salt solid electrolyte system, including polymer degradation, dehydrofluorination behavior, and their correlation with interfacial chemistry and ion‐transport characteristics. In Figure 1c, the SPE containing LiFSI shows a rapid decline in specific capacity after just a few cycles, indicating poor cycling stability. The most promising results are observed with the binary mixture of LiFSI and LiTFSI, shown in Figure 1d, resulting in a SPE that not only starts with a high specific capacity but also retains a significant portion over cycles. However, the SPE containing LiTFSI in Figure 1e maintains a relatively deteriorated capacity throughout the cycling. Figure S1 provides additional results, showing a more detailed breakdown of the cycling performance. Here, the subtle differences in cycleability among the three SPEs are further elaborated, offering a clearer picture of how the structural and electrochemical properties of each formulation contribute to their overall performance.

**FIGURE 2:**
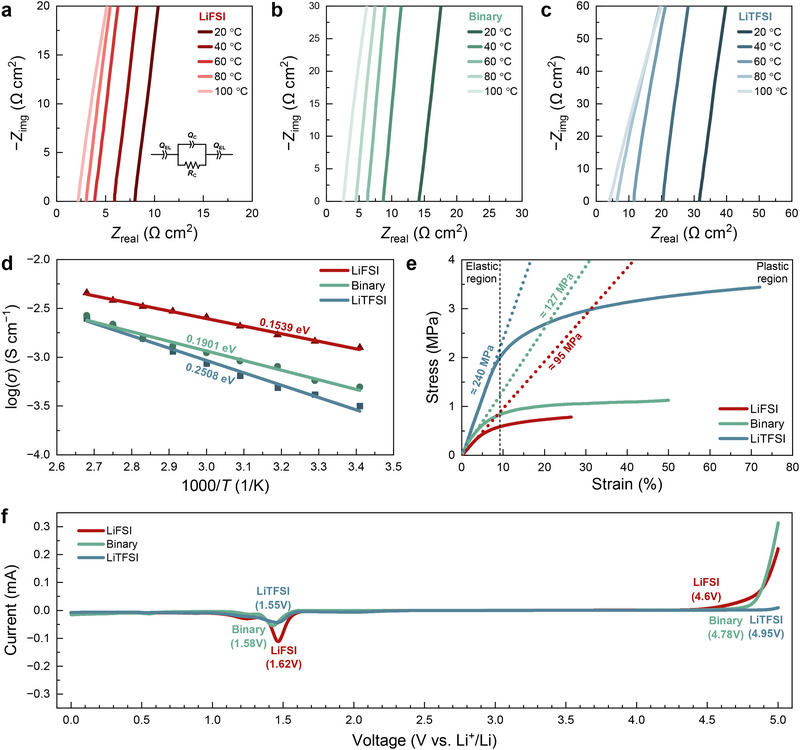
Electrochemical analysis of SPEs containing LiFSI, LiTFSI, and their binary mixture. EIS results showing the temperature dependence of ionic conductivity for SPEs with (a) LiFSI, (b) binary mixture, (c) LiTFSI, and (d) Arrhenius plot. (e) Strain‐stress curves demonstrating the mechanical properties of the SPEs. (f) LSV results the electrochemical stability window for each SPE.

To understand the phenomena during the electrochemical operation, Figure 2 presents a comprehensive electrochemomechanical analysis of the SPEs formulated with LiFSI, LiTFSI, and their binary mixture. Figure 2a–d represents the Arrhenius plots for the SPE membranes containing LiFSI, LiTFSI, and the binary mixture, respectively. The LiFSI‐based SPE, shown in Figure 2a, exhibits the lowest activation energy at 0.1539 eV, presenting the more facile energy barrier for ion transport in this system, which is not aligned to the rapid decline in cycling performance seen in earlier figures. The binary mixture, as shown in Figure 2b, presents an intermediate activation energy of 0.1901 eV, indicating a balanced performance where ionic conduction is facilitated without overly compromising the structural integrity of the electrolyte. However, the LiTFSI‐based SPE, depicted in Figure 2c, shows a higher activation energy of 0.2508 eV, suggesting relatively difficult ion migration, yet this comes at the expense of mechanical robustness. Additionally, the ionic conductivities of SPEs without SN were evaluated to investigate the sole influence of the lithium salt. As expected, the ionic conductivities of the SN‐free SPEs were lower than those with SN, consistent with the enhanced ion mobility provided by SN discussed earlier. The overall trend remained consistent, and the LiFSI‐based SPE without SN still exhibited higher ionic conductivity than both the binary‐salt SPE and the LiTFSI‐based SPE without SN. (Figure S2). This result highlights the contribution of LiFSI, whose higher Lewis basicity promotes greater dehydrofluorination of PVDF‐HFP and consequently enhances ion conduction. Both LiFSI and LiTFSI increase the population of mobile charge carriers by supplying additional lithium ions, thereby enhancing the ionic conductivity of the PVDF‐HFP–based SPE. In particular, LiFSI induces partial dehydrofluorination of the PVDF‐HFP matrix, which leads to increased segmental flexibility and reduced ion‐hopping distance. Simultaneously, the strongly electron‐withdrawing sulfonyl groups in the FSI^−^ anion facilitate ion‐pair dissociation, effectively increasing the free‐ion concentration and local charge density. The combined effects of shortened migration distance and enhanced charge density synergistically promote lithium‐ion transport and result in a significant improvement in ionic conductivity.

Figure 2e presents the strain–stress curves for the SPEs, providing insights into their mechanical properties, highlighting their elastic and plastic deformation behaviors. The LiTFSI‐based SPE demonstrates the highest modulus with 240 MPa, as evidenced by the steepest curve, which aligns with its higher crystallinity and mechanical strength. The LiFSI‐based SPE shows a much lower modulus with 95 MPa, which is consistent with its higher amorphous content and reduced mechanical integrity due to extensive dehydrofluorination [32, 33, 34]. The binary SPE exhibits a moderate modulus with 127 MPa, striking a balance between the two extremes. This balance suggests that the binary mixture can maintain sufficient mechanical stability while offering enhanced ionic conductivity, making it a more viable candidate for practical battery applications. In addition, the thermal stability of SPEs was also evaluated by TGA analysis (Figure S3). According to the results, the LiTFSI‐based SPE exhibited the highest weight retention (57.04 wt.%), compared with the LiFSI‐based SPE (40.92 wt.%) and the binary‐salt SPE (49.75 wt.%), indicating superior thermal stability. The larger weight loss observed for the LiFSI‐based SPE relative to the LiTFSI‐based SPE can be attributed to the higher weight fraction of SN in the LiFSI‐based system. Because the same molar ratio of lithium salt and SN was used, the lower molecular weight of LiFSI resulted in a higher total mass contribution from SN, thereby leading to greater weight loss during thermal decomposition. Notably, the thermal stability of the LiTFSI‐based SPE is consistent with our previous report, in which a comparable weight retention (54.1 wt.%) was observed [35], demonstrating good reproducibility of the SPE properties.

Furthermore, Figure 2f provides the linear sweep voltammetry (LSV) results, which indicate the voltage at which the SPEs begin to be electrochemically oxidized and reduced. The LiTFSI‐based SPE, with an oxidation onset potential of 4.95 V and a reduction onset potential of 1.55 V, exhibits the broadest stability window, making it particularly suitable for high‐voltage applications. In contrast, the LiFSI‐based SPE shows a narrower stability window, with oxidation and reduction onset potentials at 4.6 and 1.62 V, respectively. This limited stability window suggests that the LiFSI‐based SPE might be prone to degradation under more extreme operating conditions. The binary SPE, however, demonstrates an intermediate stability window, with onset potentials at 4.78 V for oxidation and 1.55 V for reduction, indicating that it offers a good compromise between stability and performance, further supporting its suitability for use in SSBs. Those comprehensive electrochemical analysis presented in Figure 2 exhibits the importance of combining lithium salts in the formulation of SPEs. The binary mixture of LiFSI and LiTFSI consistently demonstrates a balanced performance across ionic conduction and the mechanical robustness. Additionally, the lithium‐ion transference number was evaluated, and the binary mixture of LiFSI and LiTFSI exhibited the highest value (0.88), compared to those of the LiFSI‐based SPE (0.59) and LiTFSI‐based SPE (0.76) (Figure S4). The relatively low transference number of the LiFSI‐based SPE is attributed to the enhanced mobility of both lithium ions but also anions, which arises from the formation of highly flexible ion‐conduction pathways caused by the increased dehydrofluorination of PVDF‐HFP. In contrast, the LiTFSI‐based SPE provides a more restricted ion‐transport environment, suppressing anion mobility and thereby promoting more selective transport of the smaller lithium ions, resulting in a higher transference number than the LiFSI‐based SPE. Consequently, the binary mixture of LiFSI and LiTFSI offers an optimized balance, maintaining sufficient structural flexibility for effective lithium‐ion transport while simultaneously limiting anion diffusion, leading to the highest lithium‐ion transference number.

**FIGURE 3:**
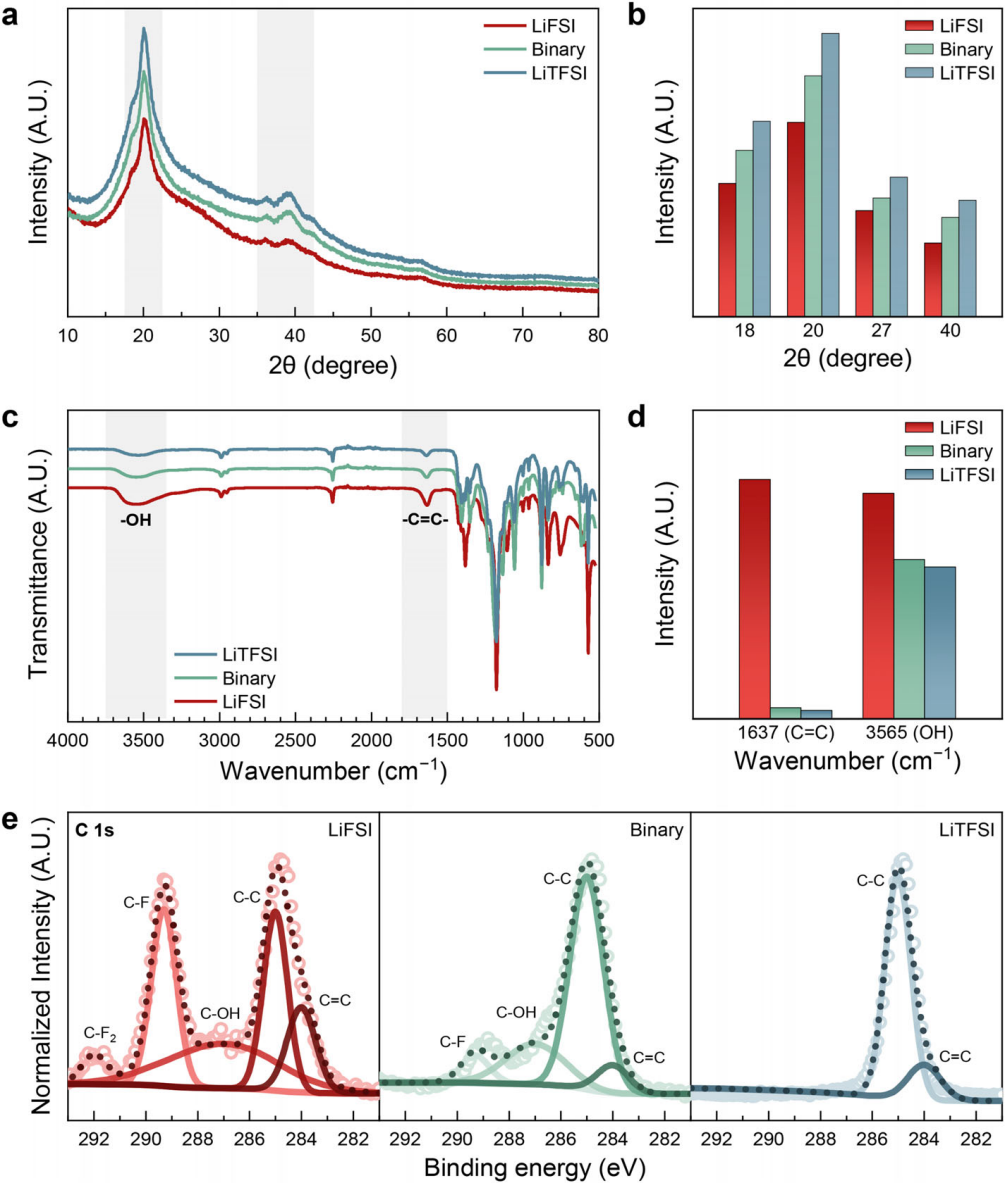
Structural and chemical characterization of SPEs. (a) XRD patterns showing the crystallinity of the SPEs. (b) Quantitative comparison of crystalline phase percentages in the SPEs, illustrating the degree of amorphization. (c) FTIR spectra highlighting the formation of C═C double bonds and OH groups. (d) Quantitative comparison of OH and C═C bonds, showing varying levels of dehydrofluorination across the SPEs. (e) XPS spectra (C 1s) confirming the presence of C═C bonds and shifts in binding energy.

Figure 3, Figures S5 and S6 offer a detailed analysis of the structural and chemical characteristics of the SPEs, particularly focusing on dehydrofluorination and its chemical consequences. Figure 3a presents the X‐ray diffraction (XRD) patterns, which reveal the crystalline structure of the PVDF‐HFP‐based SPEs [36]. The diffraction peaks correspond to different crystalline phases within the polymer matrix, and their intensities vary depending on the lithium salt used. The LiFSI‐based SPE shows a significant reduction in peak intensity, showing a higher degree of amorphization. This amorphization enhances ionic conductivity by increasing the amorphous content, which facilitates ion transport. In contrast, the LiTFSI‐based SPE retains more crystalline structure, as indicated by the sharper and more intense peaks, correlating with better mechanical properties but lower ionic conductivity as shown in Figure 2d. The binary mixture, as expected, displays intermediate characteristics, achieving a balance between ionic conductivity and mechanical strength. Figure 3b quantifies the crystalline phase percentages, further illustrating the differences among the SPEs. The LiFSI‐based SPE shows the lowest degree of crystallinity, consistent with the XRD results, while the LiTFSI‐based SPE maintains the highest crystallinity [37, 38]. The binary mixture strikes a balance, achieving crystallinity that is higher than that of the LiFSI‐based SPE but lower than that of the LiTFSI‐based one, suggesting an optimized trade‐off between ionic conductivity and mechanical strength.

The FTIR spectra in Figure 3c,d provide critical evidence of dehydrofluorination through the observation of C═C double bonds. The presence of these C═C bonds is a direct indication of dehydrofluorination, where the loss of HF from the PVDF backbone leads to the formation of unsaturated double bonds [39, 40, 41, 42]. The LiFSI‐based SPE exhibits the strongest absorption bands corresponding to these C═C bonds, confirming a high degree of dehydrofluorination. This process significantly reduces the crystallinity of the polymer, increasing the amorphous content and enhancing ionic conductivity. However, this comes at the cost of mechanical strength, as the disruption of the polymer backbone weakens the overall structure. In addition to the C═C bonds, the FTIR spectra also show the formation of OH groups, which are less commonly associated with dehydrofluorination [43, 44]. The appearance of OH groups suggests additional chemical reactions may be occurring, likely involving the interaction of liberated HF with trace moisture or oxygen, leading to the formation of hydroxyl species that are then incorporated into the polymer matrix. The LiFSI‐based SPE, with its strong Lewis basicity and reactive environment, shows the most pronounced OH peaks, expressing significant secondary reactions. The LiTFSI‐based SPE shows much weaker OH absorption, consistent with its lower reactivity and more stable structure. The binary mixture displays intermediate behavior, reflecting a balance between the reactive environment of LiFSI and the stability provided by LiTFSI.

The significance of the C═C double bonds is further reinforced in the X‐ray photoelectron spectroscopy (XPS) analysis shown in Figure 3e. The XPS spectra focus on the C 1s peaks, which are deconvoluted to reveal various chemical states of carbon, including C─C, C═C, and C─F bonds. The presence of C═C bonds in the XPS spectra provides strong confirmation of dehydrofluorination. The binding energy associated with the C═C bonds in the LiFSI‐based SPE shifts toward higher energy, implying stronger interactions within the polymer matrix and greater electron localization. This shift is a clear indicator of the structural changes caused by dehydrofluorination, where the formation of C═C bonds disrupts the original polymer structure, leading to localized electron environments that are more reactive and potentially less stable. In contrast, the LiTFSI‐based SPE shows less pronounced shifts in the C═C peak, impliciting a lower degree of dehydrofluorination and a more preserved polymer structure. The binary mixture shows an intermediate shift, suggesting that it achieves a controlled degree of dehydrofluorination, allowing for a balance between enhanced conductivity and structural integrity. Furthermore, solid‐state ^13^C NMR spectroscopy was employed to further investigate the structural changes of the SPEs (Figure S5). Compared with the LiTFSI‐based system, the LiFSI‐based SPE shows a more pronounced decrease in the C–HF‐related carbon signals, accompanied by a corresponding increase in the signals associated with C═C double bonds [45]. This indicates that the use of LiFSI promotes dehydrofluorination of the PVDF‐HFP matrix, leading to the formation of unsaturated carbon–carbon linkages.

Figure S6 presents XPS measurements of the Li metal anodes after cycling tests using SPEs formulated with LiFSI, LiTFSI, and their binary mixture. The analysis focuses on understanding the formation of key compounds, such as LiF and Li_3_N, on the surface of the Li anodes, which are critical for the stability of the solid electrolyte interphase (SEI) during cycling. In the F 1s (Figure S6a), it reveal the formation of LiF on the surface of the Li metal anodes for both the SPEs using LiFSI and LiTFSI after the cycling tests. The formation of LiF is beneficial as it is known to contribute to the stability of the SEI layer, preventing further decomposition of the electrolyte and thus enhancing the cycling performance of the battery. The presence of LiF is consistent across both SPEs, suggesting that despite the differences in the salts used, LiF formation is a common and stabilizing reaction product during cycling. However, the N 1s (Figure S6b) show a distinct difference in the chemical reactions occurring with the two salts. The Li metal anode cycled with the LiTFSI‐based SPE shows the formation of Li_3_N, a compound also beneficial for SEI stability, as it can enhance the mechanical integrity and ionic conductivity of the SEI layer. In contrast, the Li metal anode cycled with the LiFSI‐based SPE does not show significant Li_3_N formation but rather exhibits signals suggesting additional reactions that could lead to reduced electrochemical stability. These additional reactions, likely linked to the high degree of dehydrofluorination associated with the presence of nitrogen and sulfur elements in LiFSI, could introduce instabilities in the SEI layer, compromising long‐term cycling performance. Additionally, SEM/EDS analyses were performed to investigate the elemental distribution and atomic ratios of the SPEs (Figure S7 and Table S2). All constituent elements were found to be homogeneously distributed throughout the polymer matrix, indicating good miscibility and uniform incorporation of each component in the SPEs. The fluorine atomic percentages of the SPE samples were comparable; therefore, EDS analysis alone cannot provide detailed insight into the effect of lithium salt on chemical structural changes in the SPEs, particularly because it is limited to surface compositional information. Nevertheless, these results confirm the uniform distribution of all materials used in the preparation of the SPEs.

Figure S8 presents the electrochemical impedance spectroscopy (EIS) results for Li/SPEs/NCM622 solid‐state battery coin cells. The EIS measurements show that the SPE using LiFSI salt exhibits the lowest impedance, which can be attributed to the highest degree of dehydrofluorination and the resulting improved ionic conductivity of the PVDF‐HFP matrix. This low impedance is beneficial for enhancing the overall ionic conductivity of the electrolyte, contributing to better performance under certain conditions. However, the instability associated with the additional reactions identified in the XPS data suggests that while LiFSI offers superior conductivity, it may still fall short in providing long‐term stability in practical applications.

**FIGURE 4:**
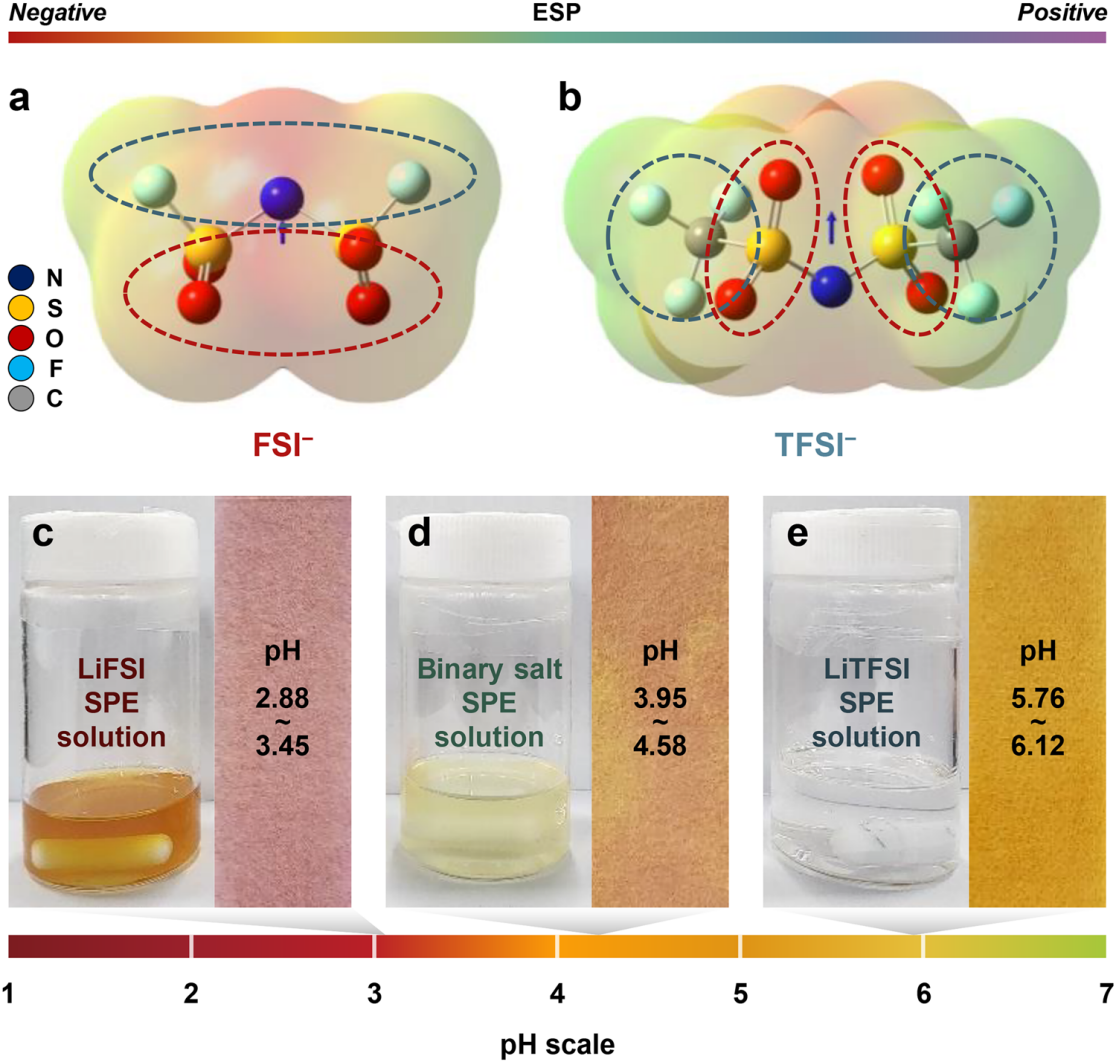
Polarity and Lewis basicity analysis of lithium salts in SPEs. (a) ESP maps of FSI^−^ and TFSI^−^ anions. (b) pH measurements of SPE solutions, showing the basicity of the salts through their interaction with the polymer matrix.

Figure 4 provides a detailed analysis of the polarity and Lewis basicity of the lithium salts used in the SPEs, which are another critical evidences influenced by the dehydrofluorination process and the overall performance of the SPEs. The polarity of the lithium salts and their basicity were assessed using electrostatic potential (ESP) maps derived from density functional theory (DFT) calculations, as well as experimental pH measurements. In Figure 4a,b, the ESP maps for the FSI^−^ and TFSI^−^ anions are presented, offering insights into the relative polarity of these anions, providing a visual representation of the electron density distribution. The FSI^−^ anion exhibits a significantly stronger negative electrostatic potential compared to the TFSI^−^ anion, as indicated by the more intense red regions in the ESP map. This stronger negative potential suggests that the FSI^−^ anion is more polar than the TFSI^−^ anion, meaning it has a higher tendency to attract positive charges, such as lithium ions. The higher polarity of the FSI^−^ anion is closely related to its stronger Lewis basicity. In general, a more polar anion can act as a stronger Lewis base because it is more effective at donating electron density to stabilize cations like Li^+^. Consequently, LiFSI, which contains the more polar FSI^−^ anion, exhibits higher Lewis basicity than LiTFSI, which contains the less polar TFSI^−^ anion. This difference in basicity is a crucial factor in the chemical behavior of the SPEs, particularly in how they interact with the polymer matrix and contribute to the dehydrofluorination process.

Figure 4c–e illustrates the experimental measurement of the basicity of the lithium salts using pH indicator. The pH values of the SPE solutions containing different lithium salts were recorded as follows: 2.88∼3.45 for the LiFSI SPE solution, 3.95∼4.58 for the binary salt SPE solution, and 5.76∼6.12 for the LiTFSI SPE solution. These pH values indicate that the solutions are acidic rather than basic. This acidity can be attributed to the dehydrofluorination process occurring within the PVDF‐HFP polymer matrix, where the removal of fluoride ions (F^−^) by the lithium salts generates HF simultaneously. The presence of HF, a strong acid, may lead to the observed acidic pH values. The correlation between the pH values and the basicity of the lithium salts can be understood in the context of the dehydrofluorination process. The stronger Lewis basicity of LiFSI accelerates the dehydrofluorination of PVDF‐HFP, leading to the generation of more HF and consequently lower pH values in the solution. In contrast, the weaker basicity of LiTFSI results in less dehydrofluorination and higher pH values. The binary mixture, which shows intermediate basicity, results in pH values that fall between those of the LiFSI and LiTFSI solutions, reflecting a moderated dehydrofluorination process. These findings, when combined with the above analysis of dehydrofluorination and chemical stability from Figure 3, clearly demonstrate the relationship between the polarity and basicity of the lithium salts and the extent of dehydrofluorination in the SPEs. The high polarity and strong Lewis basicity of LiFSI promote extensive dehydrofluorination, leading to significant structural changes in the polymer matrix, which enhance ionic conductivity but may compromise mechanical stability. On the other hand, the lower polarity and weaker basicity of LiTFSI result in less dehydrofluorination, preserving the polymeric mechanical properties but at the cost of reduced ionic mobility.

**FIGURE 5:**
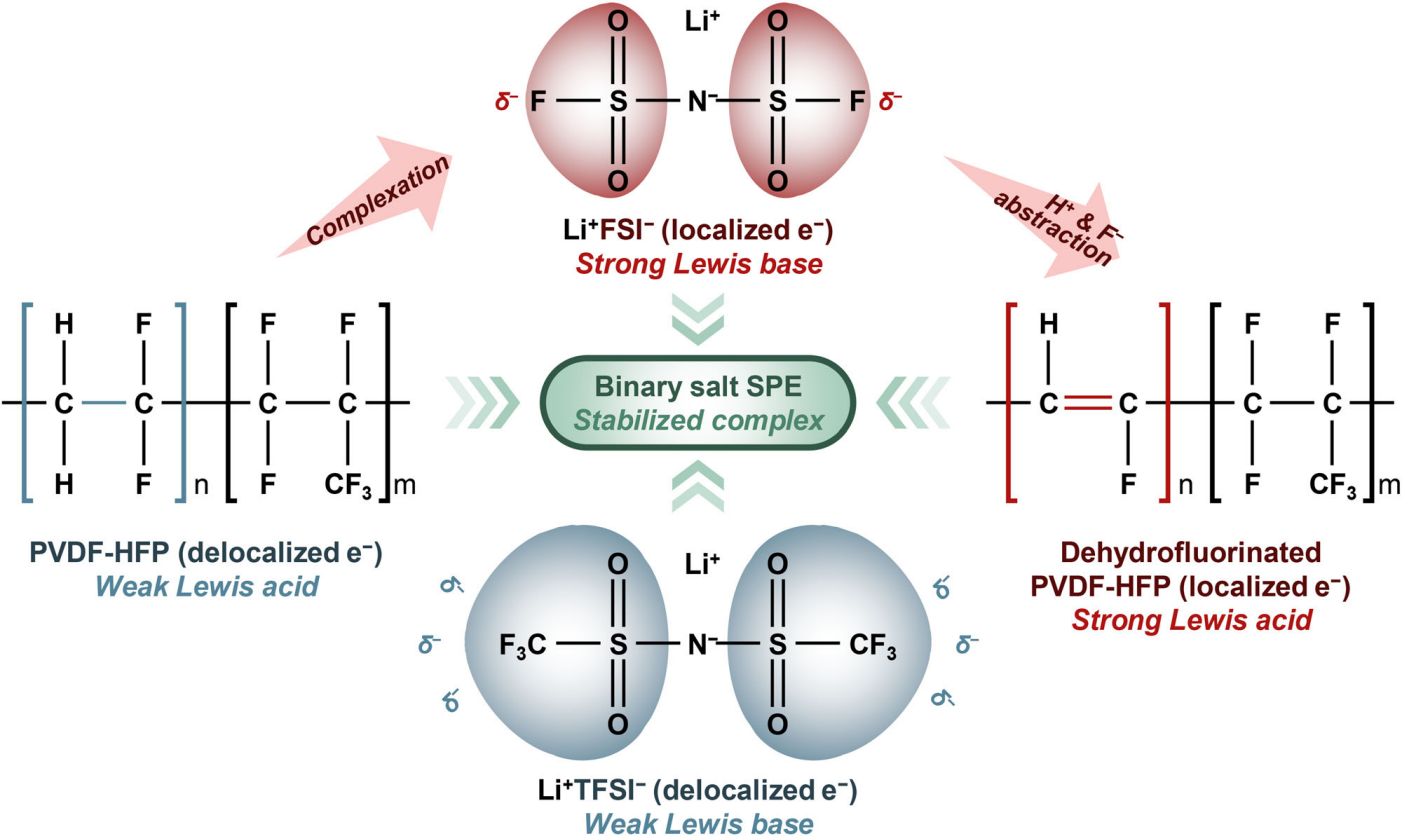
Schematic of Lewis acid‐base interactions between the PVDF‐HFP polymer matrix and lithium salts.

Figure 5 provides a mechanism illustration of the interactions between the PVDF‐HFP polymer matrix and the lithium salts used in the SPEs. It highlights the differences in how the FSI^−^ and TFSI^−^ anions interact with the polymer and the resultant effects on the dehydrofluorination process, which significantly impacts the overall stability and performance of the SPEs. The FSI^−^ anion is shown to have localized electron density, which classifies it as a strong Lewis base. This localization means that FSI^−^ can effectively interact with the electron‐rich regions of the polymer matrix. However, this strong Lewis basic behavior leads to the abstraction of proton (H^+^) and consequently fluoride ions (F^−^) from the polymer chain, driving the dehydrofluorination process. This reaction not only weakens the polymer by disrupting its structural integrity but also creates a more reactive environment, further accelerating degradation and potentially leading to instability in the SPE. However, the TFSI^−^ anion has a more delocalized electron structure, making it a weak Lewis base. Its interaction with the polymer is much less aggressive compared to FSI^−^. As a result, the TFSI^−^ anion is less likely to induce dehydrofluorination, allowing the polymer matrix to retain more of its original structural integrity. This results in better mechanical stability, albeit at the cost of lower ionic conductivity due to the reduced amorphization of the polymer. The binary salt SPE, which stabilizes the complex interaction between the polymer matrix and the anions. By combining FSI^−^ and TFSI^−^, the binary mixture balances the strong Lewis base behavior of FSI^−^ with the more moderate interactions of TFSI^−^. This combination leads to a moderated dehydrofluorination process, where the polymer matrix undergoes some degree of fluorine loss, enough to enhance ionic conductivity through increased amorphous content, but not to the extent that it severely compromises mechanical stability. The binary salt SPE thus forms a stabilized complex that optimizes both the ionic conductivity and the structural integrity of the polymer.

## 3 Conclusion

In this study, we explored the complex interactions between PVDF‐HFP and different lithium salts, LiFSI and LiTFSI, in SPEs for SSBs. By systematically analyzing the polarity, Lewis basicity, and resulting dehydrofluorination processes, we elucidated how these factors collectively influence the electrochemical performance and mechanical stability of the SPEs. Our findings demonstrate that the FSI^−^ anion, with its strong Lewis basic nature and high polarity, significantly enhances ionic conductivity by promoting dehydrofluorination and increasing the amorphous content of the polymer matrix. However, this comes at the expense of mechanical stability and long‐term electrochemical performance due to the aggressive degradation of the polymer structure. On the other hand, the TFSI^−^ anion, with its weaker Lewis basicity and lower polarity, preserves the polymeric structural integrity but limits ionic mobility due to the retention of more crystalline regions in the polymer. The introduction of a binary salt mixture of LiFSI and LiTFSI offers a promising solution, striking a balance between the competing demands of conductivity and stability. The binary salt SPE facilitates controlled dehydrofluorination, optimizing both the ionic conductivity and the mechanical robustness of the electrolyte. This balanced approach not only enhances the stability of the solid electrolyte interphase (SEI) on the Li metal anode but also improves the overall cycling performance of the battery. The combined experimental and theoretical analyses emphasize the critical role of formulating lithium salts to tailor the properties of SPEs. The insights gained from this study contribute to the broader understanding of how to design more effective and stable electrolytes for next‐generation solid‐state batteries. By optimizing the interactions within the polymer matrix, it is possible to develop SPEs that meet the requirements of high‐performing batteries.

## 4 Experimental

### 4.1 Preparation of SPE

SPEs with i) LiFSI, ii) LiTFSI, and iii) binary lithium salts were prepared with a mixture of PVDF‐HFP as polymer host, SN as a plasticizer, i) LiFSI, ii) LiTFSI, or iii) binary lithium salts (mixture of LiTFSI and LiTFSI with molar ratio of 1:1) as Li salts dissolved in Acetone as solvent. The molar ratio of SN and Li salts was 8:1, and the weight ratio of Li salts and PVDF‐HFP was 1:2. SN and Li salts were mixed and stirred at 80°C overnight, and afterward, the PVDF‐HFP and Acetone were added and stirred at 80°C overnight. The prepared SPE solutions were coated on the glass plate with the doctor blade (900 gap) and dried at 60°C for 1 h.

### 4.2 Characteristic Analysis of SPE

To measure the mechanical strength and thermal stability of the prepared SPE, dynamic mechanical analysis (DMA, TA, Q800) and thermogravimetric analysis (TGA, TA Instruments, Auto Q500) were examined, respectively. X‐ray diffraction (XRD, Smartlab (9 kW, Rigaku) was executed to identify the crystallinity of SPE. For analyzing the chemical structure of SPE, the Fourier transform infrared spectroscopy (FT‐IR, Mattson, Satelite5000), X‐ray photoelectron spectroscopy (XPS, NEXSA), and solid‐state ^13^C nuclear magnetic resonance spectroscopy (solid‐state ^13^C NMR, Bruker, Avance NEO 600) were measured. The morphology and uniformity of the prepared SPE samples were examined using scanning electron microscope/energy‐dispersive X‐ray spectroscopy (SEM/EDS, Hitachi, SU8230).

### 4.3 Preparation of Composite Electrodes Containing SPE

A cathode slurry was prepared with 94 wt.% LiNi_0.6_Co_0.2_Mn_0.2_O_2_ (NCM622, LG Chem), 2 wt.% conductive carbon black (Super P), and 4 wt.% binder solution comprising PVDF‐HFP (Sigma‐Aldrich) as the polymer host, succinonitrile (SN, > 99%, Sigma–Aldrich) as a plasticizer, and lithium bis(trifluoromethanesulfonic)imide (LiTFSI, > 98%, Sigma–Aldrich) dissolved in NMP (N‐methyl‐2‐pyrrolidinone, > 99.5%, Sigma–Aldrich). The mixture was blended in Thinky mixer at 2000 rpm for 5 min at 25°C and coated onto Al foil (99.3% purity, 20 µm thickness) using a doctor blade. It was then dried at 80°C for 12 h under vacuum.

### 4.4 Electrochemical Analysis of SPE

The electrochemical analysis was examined to identify the electrochemical properties of SPE. Electrochemical impedance spectroscopy (EIS) of stainless steel(SS)/SPE/SS cell was performed in the frequency range from 100 mHz to 7 MHz at 25°C to see the ionic conductivity of SPE. The EIS tests of SS/SPE without SN/SS cell was also performed under identical operating conditions to clearly elucidate the intrinsic contribution of the lithium salts. The lithium‐ion transference number was derived using the Bruce‐Vincent method based on chronoamperometry (CA) and EIS spectra measured before and after CA measurement of the Li/SPE/Li cell. The linear sweep voltammetry (LSV) of SS/SPE/Li cell was performed to see the electrochemical stable voltage window. All measurements were examined using Biologic (VSP).

### 4.5 DFT Calculation of LiFSI, LiTFSI, and SN

The LUMO and HOMO energies of LiFSI and LiTFSI were calculated by using Gaussian09 program to identify the oxidation and reduction stability theoretically. The vibrational frequencies were computed at DFT‐B3LYP using 3.31G** basis set.

### 4.6 Li/SPE/NCM622 Coin Cell Test

The Li/SPE/NCM622 coin half‐cell (CR2032) tests were performed with SPE using different lithium salts i) LiFSI, ii) LiTFSI, and iii) binary lithium salts. Areal cathode loading was between 2.5 to 3.5 mg cm^−2^ for tests (3.05 mg cm^−2^ for LiFSI, 3.50 mg cm^−2^ for LiTFSI, and 2.53 mg cm^−2^ for binary lithium salts). 100 µm‐thick lithium metal foil and prepared SPE were used as anode and solid electrolyte, respectively. The cathode and SPE were assembled into a membrane–electrode assembly (MEA) by warm isostatic pressing (WIP) at 5500 bar for 5 min to maximize interfacial contact between the electrode and the electrolyte. The coin cells were prepared in a dry room and tests were performed at the conditions of C‐rate of 0.1 C, cut‐off voltage range of 3.0–4.3 V at 25°C for 35 cycles using a potentiosat (WonATech, WBCS3000).

## Funding

This research was supported by a National Research Council of Science & Technology (NST) grant by the Korea government (MSIT) (No. CAP 21045‐000, No. GTL24011‐000), the Individual Basic Science & Engineering Research Program (RS‐2023‐00208929) through the National Research Foundation (NRF) of Korea funded by the Ministry of Science and ICT (MSIT), the Institute of Civil Military Technology Cooperation funded by the Defense Acquisition Program Administration and Ministry of Trade, Industry and Energy of Korean government under Grant Number. UM22213RD2, and the POSCO Science Fellowship of POSCO TJ Park Foundation (Sung‐Kyun Jung, Jinsoo Kim).

## Conflicts of Interest

The authors declare no conflicts of interest.

## Supporting information




**Supporting File**: smll72132‐sup‐0001‐SuppMat.docx

## Data Availability

The data that support the findings of this study are available on request from the corresponding author. The data are not publicly available due to privacy or ethical restrictions.
