# Supplementary material for: Lewis Acid‐Base Effects on Molecular Structure and Charge Density in Solid Polymer Electrolytes for Solid‐State Batteries
Source: Small. 2026 Jan 25;22(17):e12202. doi: 10.1002/smll.202512202 (PMC13003294; doi:10.1002/smll.202512202)
Supplement: Supplementary file 1 — Supporting File: smll72132‐sup‐0001‐SuppMat.docx [file SMLL-22-e12202-s001.docx]

**Lewis Acid-Base Effects on Molecular Structure and Charge Density in Solid Polymer Electrolytes for Solid-State Batteries**

Wonmi Lee^a,^^b.†^, Seung-Min Lee^a,c,†^, Min Kyung Kim^a,d,f,†^, Juho Lee^a,c,f^, Junhyeok Hwang^f^, Jeongsik Choi^f^, Sungbin Jang^a^, Seok Ju Kang^c,*^, Sung-Kyun Jung^e,*^, Hyun-Wook Lee^c,*^, Jinsoo Kim^a,f,*^

^a^ Ulsan Advanced Energy Technology R&D Center, Korea Institute of Energy Research, Ulsan, Republic of Korea.

^b^ Department of Chemical Engineering, Kongju National University, Cheonan, Republic of Korea

^c^ Department of Energy Engineering, School of Energy and Chemical Engineering, Ulsan National Institute of Science and Technology (UNIST), Ulsan, Republic of Korea.

^d^ Department of Nano Fusion Technology, Pusan National University, Busan, Republic of Korea

^e^ School of Transdisciplinary Innovations, Seoul National University (SNU), Seoul, Republic of Korea.

^f^ Department of Energy Science and Engineering, Daegu Gyeongbuk Institute of Science and Technology (DGIST), Daegu, Republic of Korea.

^†^ These authors contributed equally: Wonmi Lee, Seung-Min Lee, Min Kyung Kim

^*^ Email addresses of corresponding authors: jkim@dgist.ac.kr (Jinsoo Kim), hyunwooklee@unist.ac.kr (Hyun-Wook Lee), naecard@snu.ac.kr (Sung-Kyun Jung), sjkang@unist.ac.kr (Seok Ju Kang)

Figure S1. Li/SPE/NCM622 coin half-cell cycling results of SPE using LiFSI and LiTFSI with C-rate of 0.1 C, cut-off voltage range of 3.0 ~ 4.3 V at 25℃.

Figure S2. EIS results showing the ionic conductivity for SPEs without SN for (a) LiFSI, (b) binary mixture, and (c) LiTFSI.

Figure S3. TGA results showing the thermal stability for SPEs with (a) LiFSI, (b) binary mixture, and (c) LiTFSI.

Figure S4. Lithium-ion transference number for SPEs with (a) LiFSI, (b) binary mixture, and (c) LiTFSI.

Figure S5. Solid-state ^13^C NMR results for SPEs with (a) LiFSI and (c) LiTFSI.

**Figure S6.** Li metal XPS spectra of SPE using LiFSI and LiTFSI after cycling test. (a) F 1S XPS spectrat of SPE using LiFSI and LiTFSI after cycling test, (b) N 1S XPS spectrat of SPE using LiFSI and LiTFSI after cycling test.

Figure S7. SEM images for SPEs with (a) LiFSI, (b) binary mixture, (c) LiTFSI, and EDS data for SPEs with (d), (g), (j), (m) LiFSI, (e), (h), (k), (n) binary mixture, and (f), (i), (l), (o) LiTFSI while represent (d)-(f) C, (g)-(i) O, (j)-(l) F, (m)-(o) S. The scale bars are 5 μm.

**Figure S8.** EIS results of Li/SPE/NCM622 coin cell before and after cycle with utilizing SPEs containing different salts. (a) Before formation, (b) After formation.

Table S1. Comparison of resistance and ionic conductivity of SPE using different lithium salt.

Table S2. Comparison of resistance and ionic conductivity of SPE using different lithium salt.

Table S3. Comparison of properties of SPE using different lithium salt.
